# Preparation, Physicochemical Characterization, and Antioxidant Activity of Naringin–Silk Fibroin–Alginate Microspheres and Application in Yogurt

**DOI:** 10.3390/foods11142147

**Published:** 2022-07-20

**Authors:** Hongyue Wang, Hao Hu, Xindi Zhang, Lijun Zheng, Jingxin Ruan, Jiaqing Cao, Xiangrong Zhang

**Affiliations:** School of Functional Food and Wine, Shenyang Pharmaceutical University, 103 Wenhua Road, Shenyang 110016, China; wanghy19971021@126.com (H.W.); h18341440086@163.com (H.H.); zhangxindi97@163.com (X.Z.); zheng__lijun@163.com (L.Z.); rjxandzyx@163.com (J.R.); jiaqingcao@163.com (J.C.)

**Keywords:** microsphere, naringin, sodium alginate, silk fibroin, yogurt

## Abstract

Naringin is the major polyphenol in bitter orange peel with antioxidant property. However, its pH sensitivity, low solubility, and bitter taste limit its application in food. In this study, naringin–sodium alginate–silk fibroin microspheres were prepared by the ionic gel method. The loading capacity and encapsulation efficiency of naringin in microspheres were 13.2% and 77.6%, respectively. The morphology of microspheres was characterized by scanning electron microscopy. The X-ray diffractometry and differential scanning calorimetry results showed naringin was amorphous after encapsulation. Fourier-transform infrared spectroscopy and molecular docking analysis confirmed the intermolecular hydrogen bonds between naringin and sodium alginate. Naringin could release from the microspheres continuously under different pH conditions. Compared with free naringin, the 2,2-diphenyl-1-picrylhydrazyl scavenging activity and the stability of naringin microspheres were significantly improved. The application of naringin microspheres in yogurt indicated the precipitation of whey could be effectively reduced and the decline rate of pH was inhibited. The study suggested that naringin encapsulated microspheres were beneficial for improving the shelf life of this bioactive product as well as providing a new idea for functional yogurt.

## 1. Introduction

**Figure 1:**
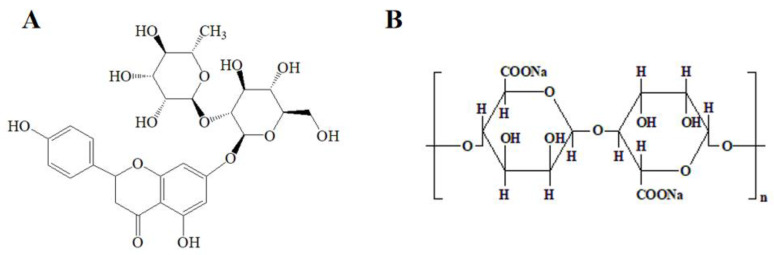
Chemical structures of (**A**) naringin and (**B**) SA.

Naringin (4′,5,7-trihydroxy flavanone 7-rhamno glucoside, Figure 1A) is a natural polyphenol compound mainly present in the peel and flesh of grapefruit, orange, and lime [1,2]. Naringin possesses a variety of biological and pharmacological properties, such as antioxidant, anti-inflammatory, antibacterial, osteoblast induction, and nerve protection [3,4,5,6]. However, its pH sensitivity, oxidability, poor solubility, and bitter flavor limit its application in food field [7]. To overcome these shortcomings, some studies investigated incorporating naringin into microcapsules, inclusion complexes, and nanoparticles [6,8,9]. Hussain et al. prepared naringin-loaded β-cyclodextrin nanoparticles to improve its bactericidal ability [6]. Nallamuthu et al. encapsulated naringin in zein/caseinate biopolymers, and the formulation had higher anti-adipogenic activity in 3T3-L1 cells comparing with the native form [10]. However, most of the work focused on examining the properties of the delivery system, and few studies investigated the stability of naringin in dosage forms. Microencapsulation is a formulation that can improve the stability of components in food and allow sustained release at specific times during food consumption [11]. As a delivery system for health products, microspheres have been applied in food products.

Sodium alginate (SA, Figure 1B) is a linear anionic polysaccharide in brown algae and one of the main components in the cell wall of sea algae [12]. SA is widely applied in encapsulation materials, which shows the characteristics of biodegradability, biocompatibility, and stability [13,14]. In addition, SA possesses a film-forming property and certain toughness and strength required by the encapsulation material; therefore, it has been widely used as an excipient in drug delivery and the food industry [15,16]. However, sometimes it is not stable enough to apply SA alone as a wall material. There are many reports on combining protein and SA to improve its stability [17,18].

Silk fibroin (SF) is the main component of silk, accounting for about 70% of the total weight. It contains 18 amino acids, including serine, alanine, and glycine [19]. SF is biocompatible, non-toxic, non-irritating, pollution free, and biodegradable. Many studies have reported the application of SF in the preparation of films, hydrogels, microcapsules, and other delivery systems [20,21,22]. The combination of SF and SA as wall materials to prepare a microsphere was feasible, and a sustained release of the encapsulated component was presented.

In this study, SA–SF–naringin microspheres were fabricated by the ionic gel method. The microspheres were characterized by scanning electron microscopy (SEM), X-ray diffractometry (XRD), and differential scanning calorimetry (DSC). Fourier-transform infrared spectroscopy (FTIR) and the molecular docking technique were employed to explain the molecular interaction between SA and naringin. The in vitro release, 2,2-diphenyl-1-picrylhydrazyl (DPPH) radical scavenging activity, and storage stability of microspheres were investigated. Finally, the microspheres were applied to yogurt, and their effects on the pH, water holding capacity, and aroma were evaluated.

## 2. Materials and Methods

### 2.1. Materials

Naringin was purchased from Shanghai Aladdin Biochemical Technology Co., Ltd. (Shanghai, China). Ethyl oleate was obtained from Shanghai Yuanye Biotechnology Co., Ltd. (Shanghai, China). Tween 80 and Magnesium stearate were supplied by Tianjin Damao Chemical Reagent Factory (Tianjin, China). SF was purchased from the Shandong Qilu Biotechnology Group Co., Ltd. (Liaocheng, China). SA was acquired from Henan Wanbang Chemical Technology Co., Ltd. (Zhengzhou, China). Calcium chloride was obtained from Tianjin Hengxing Chemical Reagent Manufacturing Co., Ltd. (Tianjin, China). All other chemicals and reagents were of analytical grade.

### 2.2. Preparation of Microspheres

**Figure 2:**
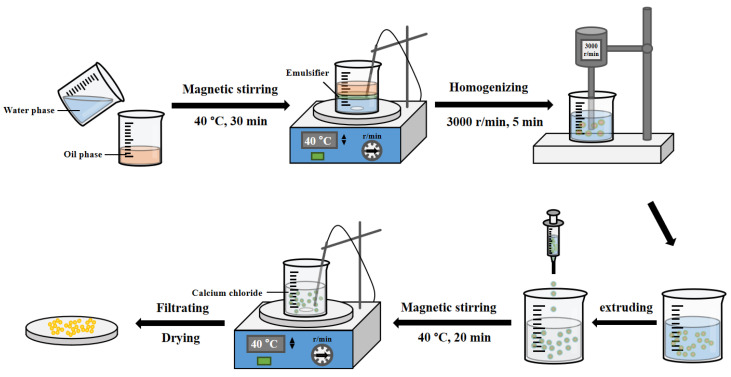
Schematic diagram of the production of microspheres.

Naringin was encapsulated using the emulsion gelation method reported by Huang et al. [23] with some modifications (Figure 2). Briefly, appropriate amounts of magnesium stearate, Tween 80, and naringin powder were added to ethyl oleate and mixed under magnetic stirring for 10 min at 40 °C to form an oil phase. SA was put into 25 mL of deionized water and stirred at 60 °C for 30 min to ensure it completely dissolved. Then, the temperature was kept at 40 °C and SF was added and mixed as the aqueous phase. The aqueous phase was dropped into the oil phase under magnetic stirring at 40 °C for 30 min to form the coarse emulsion. Then, the emulsion was homogenized for 5 min at 3000 r/min with a high shear force dispersion emulsifier (FM200, Shanghai Fluke Technology Development Co., Ltd., Shanghai, China) to obtain the emulsion. The emulsions were extruded at a uniform speed drop by drop using syringes with a #30G needle into the calcium chloride solution and incubated at 40 °C for 20 min. Microspheres were removed by suction filtration and rinsed twice with deionized water to remove the unencapsulated naringin powder. Finally, the microspheres were dried at 25 °C for 12 h and stored in a desiccator until future analysis.

### 2.3. Determination of Encapsulation Efficiency (EE), Loading Capacity (LC), and Particle Size

In order to determine the EE and LC, a certain number of microspheres were weighed. Then, they were ground into powder with a mortar and dissolved in methanol. The concentration of naringin was measured by an HPLC (L-7110, Hitachi Instruments Co., Ltd., Tokyo, Japan) at 282 nm. The mobile phase was methanol–water–acetic acid (42:54:4, *v*/*v*/*v*) at a flow rate of 1.0 mL/min. The method was performed on the C_18_ column (Diamonsil 250 × 4.6 mm) with an injection volume of 20 μL. The concentration of naringin was calculated according to the standard curve. The equations to calculate EE% and LC% were as follows:

$$\mathrm{EE}\%=\frac{\mathrm{Total}\;\mathrm{amount}\;\mathrm{of}\;\mathrm{loaded}\;\mathrm{naringin}}{\mathrm{Initial}\;\mathrm{amount}\;\mathrm{of}\;\mathrm{naringin}}$$

$$\mathrm{LC}\%=\frac{\mathrm{Total}\;\mathrm{amount}\;\mathrm{of}\;\mathrm{loaded}\;\mathrm{naringin}}{\mathrm{Microspheres}\;\mathrm{amount}}$$

The particle size of naringin-loaded microspheres was measured by Nano Measurer 1.2 software. Three batches of microspheres were prepared, each with more than 100 microspheres, and they were photographed separately. The scale was determined in the software. Then, each microsphere was labeled and the diameter was determined.

### 2.4. Scanning Electron Microscopy

The morphology of naringin-loaded microsphere and the cross section were observed by an SEM (Merlin, Carl Zeiss AG, Oberkochen, Germany). The cross section was obtained by severing. The microspheres were coated with platinum in a sputtering coater and then observed and photographed. 

### 2.5. X-ray Diffractometry

XRD values of the SA, naringin, physical mixture (PM), and microspheres were determined using an X-ray diffractometer (D8, Bruker Instruments Co., Ltd., Billerica, MA, USA) with a copper anode handled at Cu Kα radiation (1.5406 A, 40 kV, and 30 mA) after passing through nickel filters and were analyzed within a 2θ scale from 5 to 60° at a rate of 4°/min.

The physical mixture was obtained by mixing the SA, SF, and naringin in the same amount as the microspheres.

### 2.6. Differential Scanning Calorimetry

The thermodynamic analysis of the SA, naringin, PM, and microspheres was performed by differential scanning calorimetry (DSC 25, TA Discovery, Newcastle County, DE, USA). Indium was used to calibrate the temperature and enthalpy. Samples (approximately 3–5 mg) were weighed in sealed aluminum crucibles and heated from 20 to 300 °C at a rate of 10 °C/min in a nitrogen flow of 50 mL/min with an empty crucible as reference.

### 2.7. Fourier-Transform Infrared Spectroscopy

The FTIR spectra of the SA, naringin, PM, and microspheres samples were recorded on an FTIR spectrophotometer (VETEX80, Bruker Instruments Co., Ltd., Billerica, MA, USA) using the potassium bromide pressed disk technique in the range of 4000–500 cm^−1^ at ambient temperature.

### 2.8. Molecular Docking

The structures of the SA and naringin were generated by ChemDraw 16.0 software (PerkinElmer, Inc., Waltham, MA, USA). To establish the 3D structure of the SA, the minimization and kinetic functions of the structure were optimized using Sybyl 6.9.1 software (Tripos Associates Inc., St. Louis, MO, USA). The optimization parameters were the 10,000 of max iterations and the 0.005 kcal/mol of energy change, which were minimized by the Powell method (Tripos force field). The charge was distributed by the Gasteiger–Huckel method with an energy change of 0.005 kcal/(mol·A) and the remaining parameters remained the same. The docking interaction of SA–naringin was predicted using AutoDock 4.2 software (Olson Lab, TSRI, San Diego, CA, USA). The number of generations and individuals for molecular docking was 100, and the maximum number of energy evaluations was 25,000,000 per run. The lowest energy conformations generated by AutoDock were considered the best binding conformations between SA and naringin.

### 2.9. Solubility and In Vitro Release Studies

In order to measure the solubility, naringin and microspheres were put into 10 mL of deionized water, respectively, and stirred for 48 h at 25 °C. These solutions were filtered and analyzed by an HPLC (L-7110, Hitachi Instruments Co., Ltd., Tokyo, Japan).

The in vitro release of naringin from microspheres under pH 1.2, 4.5, 6.8, and 7.4 and water was performed in the dissolution apparatus (RC-6, Tianjin Shengdasanhe Optical Instrument Co., Ltd., Tianjin, China) according to a previously reported method with some modifications [24]. The composition of the dissolution medium is shown in Table S1. The choice of pH value was mainly based on the simulation of pH in the gastrointestinal tract to investigate the release of naringin. Approximately 1 g microspheres were placed in a rotating basket with 900 mL of dissolution medium and stirred at 100 rpm at 37 °C. The dissolution medium was collected at predetermined time intervals, and an equal amount of fresh dissolution medium was immediately replaced. The collected dissolution media were filtered through a 0.45 μm filter and then analyzed by an HPLC (L-7110, Hitachi Instruments Co., Ltd., Tokyo, Japan) to quantify the amount of naringin. The equation to calculate the cumulative release rate is as follows:

$$Cumulative\;release\;rate\;\left(\%\right)=(C_{n}V_{0}+V_{S}\overset{n}{\underset{i-1}{\sum }}C_{i-1})÷W\times 100\%\;\;\;\;\;\;\;\;i=1,2,3\cdots$$

where *C_n_* is the concentration of naringin in the release medium measured at the *n*-th sampling, *V*_0_ is the total volume of the medium, *V_s_* is the sampling volume, and *W* is the mass of naringin contained.

The obtained release results from this study were fitted to kinetic models including zero-order, first-order, and Higuchi’s matrix by Origin Pro2021 software (OriginLab, Northampton, MA, USA). The relevant correlation coefficients were taken into consideration to select the best model.

### 2.10. DPPH Radical Scavenging Activity

The antioxidant activity of naringin and microspheres was assessed by the DPPH radical scavenging ability according to a previously reported method with some modifications [23]. The color of DPPH solution turns lighter in the presence of antioxidants and results in a change of absorbance. The different absorbances determine the antioxidant capacity [25]. Briefly, 0.4 mL of each sample (naringin or microspheres powder in 80% methanol) was added to 0.6 mL of DPPH ethanol solution, and the absorbance was measured at 517 nm using a spectrophotometer (UV-5100, Shanghai Jinghong Laboratory Instrument Co., Ltd., Shanghai, China) after reaction in the dark for 30 min. The DPPH free radical scavenging rates of samples were calculated according to the following equation:

$$DPPH\;free\;radical\;scavenging\;rate\;\left(\%\right)=\left(1-\frac{A_{sample}-A_{control}}{A_{blank}}\right)\times 100$$

where *A_sample_* is 0.4 mL of sample and 0.6 mL of DPPH solution, *A_control_* is 0.4 mL of sample and 0.6 mL of 80% methanol, and *A_blank_* is 0.4 mL of 80% methanol and 0.6 mL of DPPH solution.

### 2.11. Stability Study

The appearance and retention rate of naringin and microspheres were investigated under four distinct storage environments, including high temperature (60 °C, 75% relative humidity (RH)), high humidity (25 °C, 95% RH), illumination (25 °C, 4500 lx), and storage (40 °C, 75% RH) using a drug stability apparatus (WD-2A, Tianjin Jingtuo Instrument Technology Co., Ltd., Tianjin, China). The samples were photographed at predetermined time intervals, and the contents were measured according to the method in Section 2.3. The retention rates were also determined.

### 2.12. Preparation of Yogurt

The pectin (3 g) was put into 400 mL of water and left overnight to fully dissolve. Then, aspartame (0.3 g), chicory fructooligosaccharide (3 g), and 500 mL of reconstituted milk (100 g of skim milk powder dissolved into 500 mL water) were added the next day. The solution was heated to 90 °C for 5 min and rapidly cooled to 45 °C. Then, the bacterial powder was added and packaged into boxes. The fermentation was at 40 °C for 7.5 h in a yogurt fermenter (SN01, Henan Xifan Electric Appliance Co., Ltd., Shangqiu, China).

After fermentation, the yogurt was stirred until there were no particles, and we took out a certain amount of yogurt. The free naringin (0.7%) or naringin microspheres (5.0%) were added to the yogurt and mixed for some time. The control yogurt (CY) was the sample without adding naringin or microspheres. The obtained yogurt was left at 4 °C for 15 h in order to perform post-fermenting.

### 2.13. Characterization of Yogurt

#### 2.13.1. Determination of pH and Water Holding Capacity

The pH value and water holding capacity of yogurt were measured on days 1, 7, and 14, respectively. The pH value was measured using an acidity meter (Light Magnetic PHS-3C, Shanghai Yidian Scientific Instrument Co., Ltd., Shanghai, China). 

The pictures of prepared yogurts were taken on days 1, 7, and 14, and the water holding capacity of the three yogurts was determined according to the degree of whey precipitation.

#### 2.13.2. Analysis of Aroma

Yogurts were put into a headspace vial and heated at 40 °C for 10 min. Then, two needles were put on the top of the headspace vial. One was connected to the environment, and the other was connected to the electronic nose. The measurement was performed using an electronic nose (PEN3, Airsense Analytics, Schwerin, Germany) with the parameters of sample interval 1 s, zero point trim time 10 s, pre-sampling time 5 s, chamber flow 300 mL/min, initial injection flow 300 mL/min, and measurement time 120 s. The results of the aroma of yogurt were analyzed by Winmuster 1.6.2 software (Airsense Analytics, Schwerin, Germany). 

### 2.14. Statistical Analysis

All the tests were performed at least in triplicate using a completely randomized design. The results are presented as mean ± standard deviation (SD). One-way ANOVA was used to assess significant differences at the 0.05 probability level.

## 3. Results and Discussion

### 3.1. Preparation of Microspheres

EE% and LC% are parameters to evaluate the performance of microspheres, which can reflect the encapsulation [26]. According to the results of EE% and LC%, the low concentration of SA led to the low EE% and LC%. The reason was that the carboxyl group of the alginate could bind to calcium ions. Once the binding site was fully occupied, the excess calcium ions would no longer be able to merge into alginate microspheres [23]. Similarly, a high concentration of calcium chloride possessed the same effect. When the concentration of SA or SF was over 15 and 50 mg/mL, respectively, the emulsion could not be extruded from the needle, leading to the irregular shape of microspheres because of the high viscosity of the emulsion. Ruan et al. also found similar results [18]. Finally, the mass ratio of SA, SF, and naringin was 1:4:4, the oil–water ratio was 1:5, and the concentration of calcium chloride solution and SA solution was 50 mg/mL and 10 mg/mL, respectively. Tween 80 was selected as an emulsifier and magnesium stearate was selected as a dispersant to prevent adhesion between microspheres. According to the reproducibility results between batches, the mean values of EE% and LC% were 77.6% and 13.2%, respectively (*n* = 3). 

According to the results of the microspheres’ particle size, the diameter of microspheres was found in the range of 200 μm to 800 μm. More than 88.7% of the total microspheres were between 400 μm and 700 μm in size, and about 36.0% with the particle size of 500 μm to 600 μm.

### 3.2. Scanning Electron Microscopy Analysis

**Figure 3:**
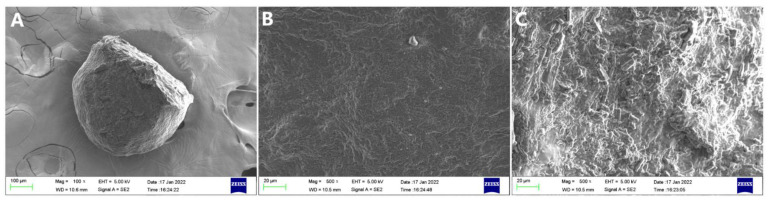
SEM image of (**A**,**B**) appearance of microsphere, (**C**) cross section of microsphere.

The SEM images of microspheres are shown in Figure 3. The microsphere was spherical and rough with tight pores on the surface (Figure 3A, 100× magnification), which might have been the dense structure formed by the cross-linking of SA with the SF protein [27]. Some studies also showed similar images, which may have been caused by the use of SA and the same microspheres’ development methods [18,23]. The SEM image of the formulation with crosscut was taken at 500× magnification, as shown in Figure 3C. The cross section of the microsphere was similar to the surface structure (Figure 3B, 500× magnification); the tighter surface structure compared to the cross section may have been due to cross-linking with calcium chloride [28]. In general, the overall structure of the microspheres was uniform. 

### 3.3. Differential Scanning Calorimetry

**Figure 4:**
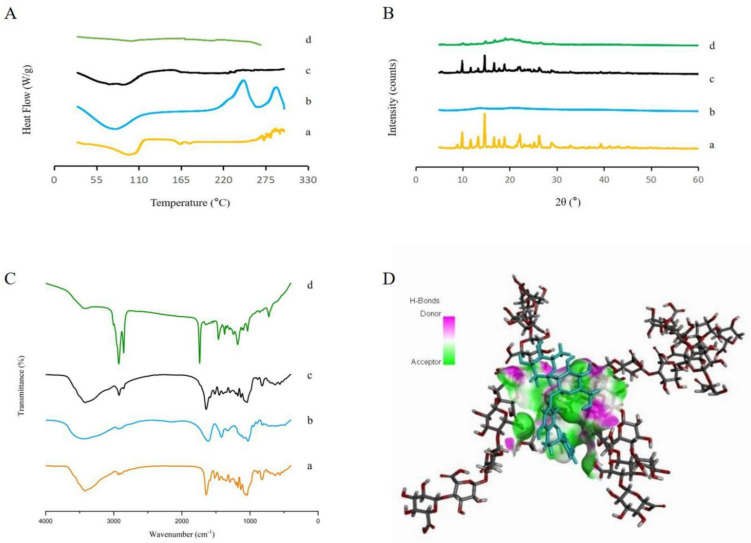
Characterizations of (**A**) DSC thermograms, (**B**) XRD patterns, and (**C**) FTIR spectra. Naringin (a), SA (b), PM (c), and microspheres (d). (**D**) The 3D conformations of SA and naringin by molecular docking (hydrogen bonding in green, O atoms in red, C atoms in gray, and naringin in blue).

DSC thermograms of the SA, free naringin, PM, and microspheres are shown in Figure 4A. Naringin showed a dehydration band at 98 °C and an endothermic peak at 164 °C, associated with the melting point of crystalline naringin [29]. For the SA, the endothermic peak appeared at 78 °C, which could be attributed to the dehydration process or the melting of SA; an exothermic peak at approximately 250 °C, referred to as the melting temperature, and chemical changes also took place at this temperature [30]. Compared with the free naringin powder, the PM still had dehydration bands under 100 °C. The endothermic peak of naringin was concealed in the PM, which could be explained by the low proportion of naringin and the dissolution of melt SA. For microspheres, the absence of melting of the naringin and the SA possibly indicates its amorphous nature.

### 3.4. X-ray Diffractometry

According to Figure 4B, the naringin showed sharp diffraction peaks, revealing its crystalline state, which is similar to the result obtained by Xiang et al. [31]. As an amorphous excipient, the SA did not show sharp crystal diffraction peaks. This phenomenon was also observed by Essifi et al. [32]. The naringin diffraction peaks could still be observed in the PM, indicating the crystalline structure of naringin was not changed. There was only a minor number of diffraction peaks in the microspheres, suggesting that most of the naringin was encapsulated in an amorphous state. The results were consistent with those of the DSC. 

### 3.5. Fourier-Transform Infrared Spectroscopy

The spectra of the SA, free naringin, microspheres, and PM are shown in Figure 4C. The SA showed a broad and intense peak at 3435 cm^−1^, which was attributed to the O−H stretching. The peaks at 1613 and 1419 cm^−1^ were due to asymmetrical and symmetrical vibrations of COO−. The peak at 1030 cm^−1^ corresponded to the C−O stretching vibration of the pyranose ring and the peak at 950 cm^−1^ was due to the C−O stretching, with contributions from C−C−H and C−O−H deformation [33]. The characteristic absorption peaks of naringin were present, including 3424 cm^−1^ (stretching vibration of O−H), 2889 cm^−1^ (asymmetric stretching vibration of methyl), 1644 cm^−1^ (stretching vibration of C=O), 1585 cm^−1^, 1516 cm^−1,^ and 1450 cm^−1^ (benzene ring) [34]. Several changes in the peaks indicated intermolecular interactions between SA and naringin after the microencapsulation. The stretching of the hydroxyl groups in the microspheres disappeared compared with the SA, free naringin, and PM, indicating the hydroxyl groups’ hydrogen donation. The intense peak of C=O at 1644 cm^−1^ in the naringin disappeared; a new peak appeared at the wave number of 1739 cm^−1^, which was probably the formation of –COOH. The SA and naringin contained O−H and C=O as hydrogen donors and acceptors, respectively. These intermolecular interactions explained the stabilization of the microspheres.

### 3.6. Molecular Docking

The molecular docking methods provided information to predict the interaction between the SA and naringin at a molecular level. The molecular docking experiments were an excellent complement to the FTIR results because of the complexity of the infrared spectrum [35]. After optimization, both the SA and naringin with the lowest total energy were formed. The minimum energy complex of SA–naringin (Figure 4D) showed that the C=O group of the SA formed hydrogen bonds with the O–H group of the naringin. The binding energy value of SA–naringin was –3.74 kcal/mol, indicating the formation of the naringin–SA microspheres was a stable thermodynamics process. The above results were consistent with the results of the FTIR.

### 3.7. Solubility and In Vitro Release Studies

The aqueous solubility of encapsulated naringin (1.01 ± 0.04 mg/mL, *n* = 3) was increased compared with free naringin (0.85 ± 0.02 mg/mL, *n* = 3). This can be explained by the transition from a crystal to an amorphous state; the results were consistent with the DSC and XRD [36]. Kim et al. encapsulated revaprazan into a gelatin microsphere and its solubility improved significantly [37]. Hu et al. also reported the same result, which incorporated curcumin by the cross-linking of sodium alginate with calcium chloride [38].

**Figure 5:**
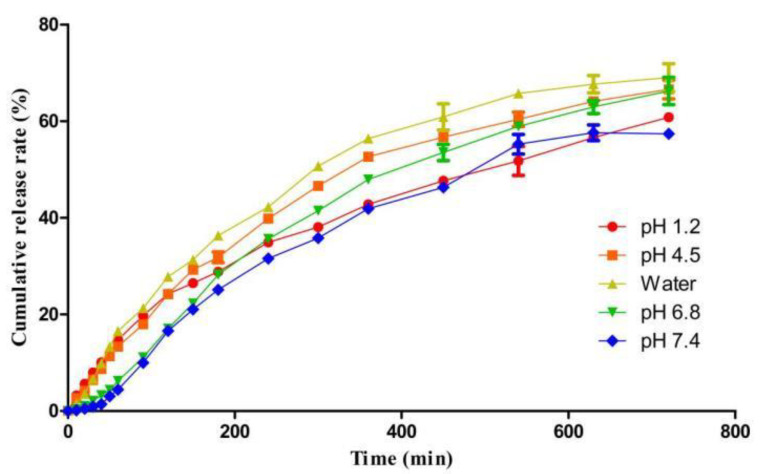
Effect of various pH dissolution media on naringin release behavior.

The release profiles of the naringin from the microspheres with different pH values were compared, as shown in Figure 5. With the increase in pH, the cumulative release rate of the naringin showed a trend of first increasing and then decreasing. The reason could be interpreted as that under the lower pH condition, carboxyl groups in alginate were converted to acid, leading to the formation of alginic acid [39], and hindered the release of the naringin from the microspheres, while at a higher pH (pH 6.8 and pH 7.4), the alginate was soluble and naringin was released by both diffusion and erosion from alginate. The dissolved alginate adhered to the surface of the microspheres and prevented the diffusion of naringin from the dissolution with a decrease in the cumulative release rate. Hussain et al. prepared naringin-β-cyclodextrin nanoparticles and obtained, through in vitro study, that the maximum release of naringin was 81.3% at pH 7.0 and only 53.8% at pH 4.6. The maximum release time was 8.5 h under both pH conditions [6]. In our research, the release curves at various pH values were about similar and the cumulative release of naringin was up to 60% during 12 h. 

As seen from Table 1, all release models conformed to the first-order kinetic model (R^2^ > 0.98) except for pH 7.4. Therefore, it was fitted to the Ritger–Peppas equation (*Mt* = 0.39*k*^0.77^, R^2^ = 0.9691). According to the Ritger–Peppas equation, when *n* value was less than 0.45, the release mechanism was Fickian diffusion; when the *n* value was between 0.45 and 0.89, the release mechanism was non-Fickian diffusion, indicating diffusion and skeleton dissolution acted together; and when the *n* value was more than 0.89, the release mechanism was skeleton dissolution [40]. In our study, the *n* value was 0.77 at pH 7.4, which indicated that diffusion and skeleton dissolution acted simultaneously.

### 3.8. DPPH Radical Scavenging Activity

**Figure 6:**
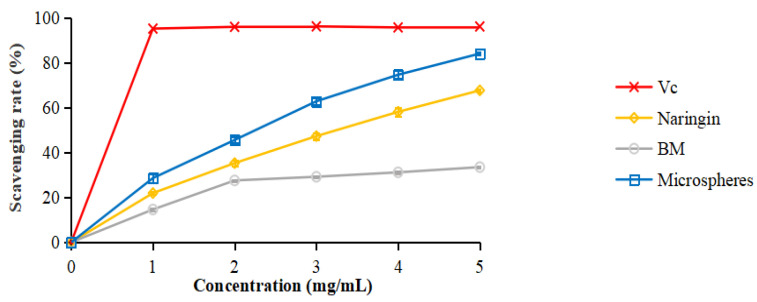
DPPH radical scavenging activity of different concentrations of Vc, naringin, BMs, and microspheres.

The DPPH radical scavenging activity of the naringin and microspheres was evaluated as an antioxidation ability. As shown in Figure 6, the scavenging rate of the samples significantly increased as the concentration increased from 1 to 5 mg/mL. The free naringin scavenging rate was 67.7%, while that of the microspheres was 84.1% compared with ascorbic acid (Vc) and 96.2% in the concentration of 5 mg/mL. They were both significantly higher than that of free naringin. The reason was that there were antioxidant components in the wall materials of the microspheres [41]; the same result was seen in the curve of the free radical scavenging activity of the blank microspheres (BMs). The BMs were prepared in the same way as the naringin microspheres without adding naringin. The scavenging rate of the microspheres kept increasing with the increase in concentration. It was predicted that they might reach or exceed the level of Vc at the concentration of 6.3 mg/mL. However, this needs future research. Therefore, the encapsulation of the naringin into the microspheres possessed a strong antioxidant capacity and improved the antioxidant potential in functional food development.

### 3.9. Stability Study

**Figure 7:**
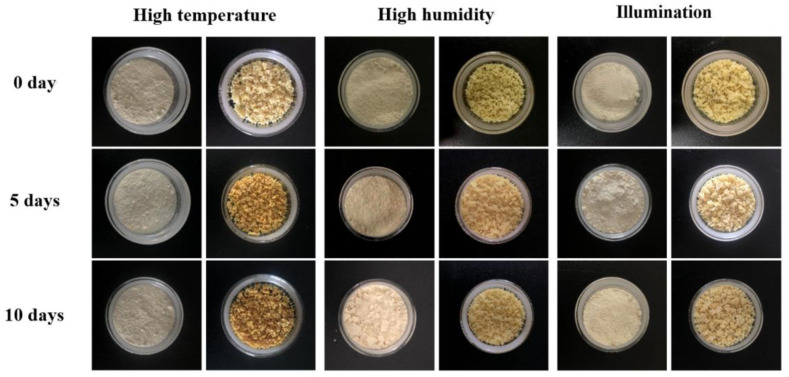
The change of free naringin and microspheres under different conditions on days 0, 5, and 10.

The retention rate of naringin is an important indicator to measure the storage stability of the microencapsulated naringin. As shown in Table 2, the retention rate of the free naringin was significantly reduced under the conditions of high temperature or humidity or illumination for 10 days, while the encapsulated naringin only decreased 2.5%, 6.2%, and 5.3%, respectively, compared with day 0. This phenomenon was also observed after 3 months (Table 3), when the retention rates of the free naringin and microspheres were 79.4% and 88.8%, respectively. Huang et al. reported that the residual content of lutein in the microspheres under various conditions was more than that of free lutein at 60 days [23]. As shown in Figure 7, the darker color of the microspheres might have been induced by the decreased water content of the microspheres at high temperature. The free naringin agglomeration was observed except at high temperature due to the hygroscopicity of the naringin powder. These results indicated that the encapsulation had a protective effect on naringin and that the microspheres could keep the naringin stable in long-term storage. Ruan et al. also showed a similar result, i.e., that the color, content, and retention of tea polyphenol in microspheres had no apparent changes under sealed, dry, and dark conditions for half a year [18].

### 3.10. Characterization of Yogurt

**Figure 8:**
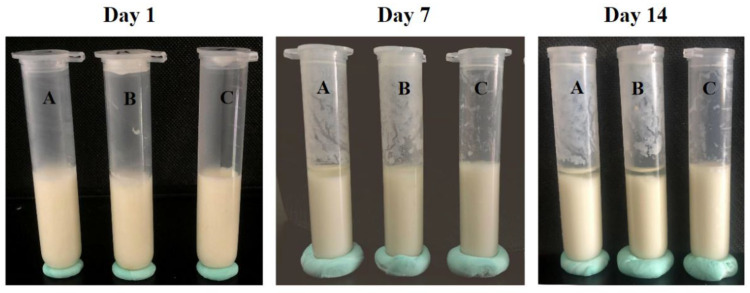
The whey precipitation of yogurt (A: CY, B: NY, C: MY).

As was already known, some whey or water in yogurt could separate during storage. By comparing the degree of whey precipitation for the three kinds of yogurt on days 1, 7, and 14, as shown in Figure 8, the phase separation was not evident in yogurt on the first day. On days 7 and 14, only a small amount of whey could be seen in the yogurt with microspheres (MY), which was significantly less than that in CY and yogurt with naringin (NY). This result might have been caused by the combination of the SA and SF in the microspheres, which made the accumulation clusters formed by casein particles in the yogurt connect to form a dense, uniform, and firm chain spatial structure [42]. Sun et al. reported the same result when they added cinnamaldehyde-modified whey protein-stabilized microcapsules into yogurt and inhibited the separation of whey solution [43].

In storage, the acidity of yogurt would increase gradually due to microbial acid production, but high acidity would produce a stimulating taste and inhibit beneficial bacteria that do not tolerate acid in the intestine. As shown in Table 4, the pH of yogurt decreased during the storage, and there was no significant difference between the pH of NY and CY on days 7 and 14. The pH value of the microspheres was always higher than that of the other two groups and decreased at a slower rate, which might have been due to the antibacterial activity of naringin and microencapsulated, sustained release properties [6].

**Figure 9:**
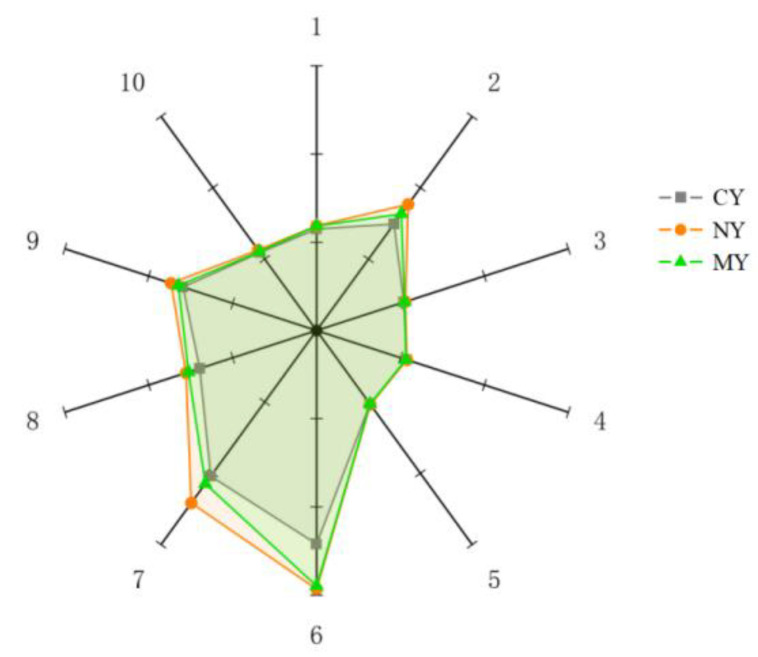
Aroma radar map of different yogurts.

The aroma radar plot of yogurt is presented in Figure 9. The R6 and R7 sensor response values were higher for all three yogurts, indicating that alkanes and sulfides were more present in the yogurt. The R2 (nitrogen oxides), R6 (methane), R7 (sulfides), and R8 (alcohol) sensor response values increased for NY and MY compared to CY, while MY increased less than NY, indicating that microencapsulation contributed to inhibiting the effect of the naringin aroma on the flavor of yogurt.

**Figure 10:**
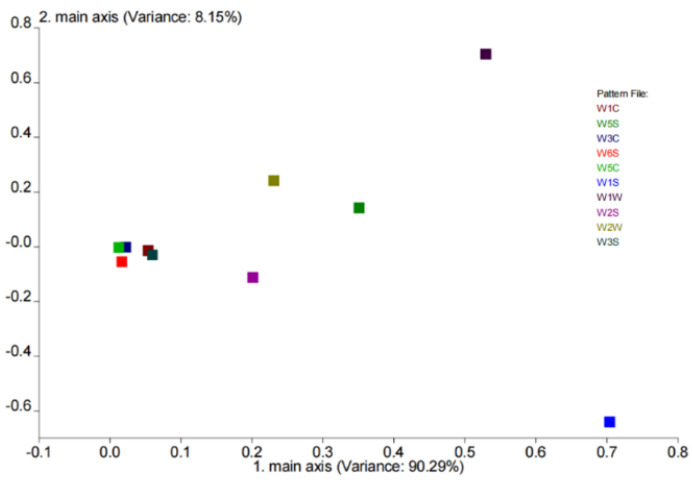
The loadings’ analysis of different yogurt.

According to the results of the loadings’ analysis (Figure 10), the coordinates of the W1S sensor were farthest from *x* = 0, indicating that W1S (methane) contributed the most to the first principal component, while the coordinates of the W1W sensor were farthest from *y* = 0, suggesting that W1W (sulfides) contributed the most to the second principal component.

**Figure 11:**
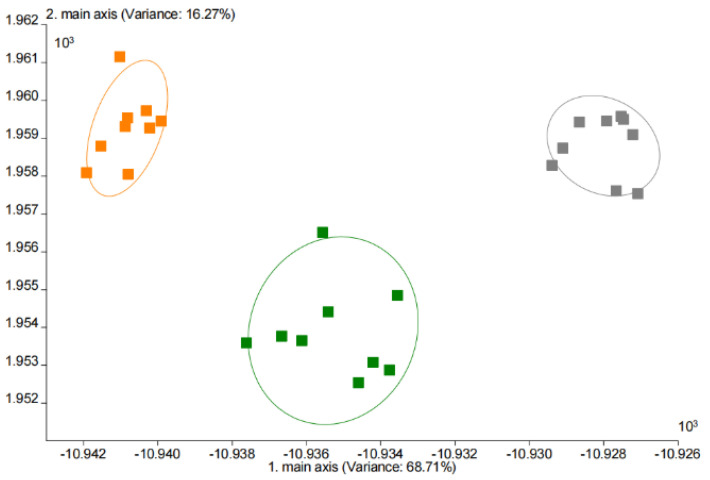
The LDA analysis of different yogurts (CY in gray, NY in orange, and MY in green).

The LDA analysis of the aroma components of the yogurt is shown in Figure 11. The first principal component contributed 68.7% and the second contributed 16.3%, giving a total contribution of 85.0%, which was a good representation of the three yogurt aromas. MY was closer to CY in comparison, indicating that the aroma of MY was more relative to CY than to NY, which was consistent with the results of the aroma radar plot.

## 4. Conclusions

The present study successfully fabricated SA–SF–naringin microspheres by the ionic gel method. The XRD and DSC analyses showed significant reduction in naringin crystallinity after it was incorporated into the SA. Based on the FTIR and molecular docking, hydrogen bonding formed between the hydroxyl groups of the naringin and the carbonyl groups of the SA. The in vitro release of naringin from the microspheres under pH 7.4 conformed to non-Fickian diffusion. Microencapsulation also enhanced the DPPH radical scavenging activity and the stability of naringin compared with free naringin. The microspheres could effectively reduce whey release in yogurt, inhibit the pH decline rate, and mask the taste of the naringin. Therefore, the fabricated microspheres might be applied to the production of functional food. This study provides a new approach for preparing naringin health products and their application in functional and nutritional yogurt.

## Figures and Tables

**Table 1:** Model simulated for the release profiles of microspheres within 0–12 h.

	Zero-Order		First-Order		Higuchi	
pH	Equation	R^2^	Equation	R^2^	Equation	R^2^
1.2	*M_t_* = 0.09 *t* + 8.70	0.9312	*M_t_* = 62.51(1 − e^(^^3×10^(−3))t^)	0.9912	*M_t_* = 2.43*t*^1/2^ − 3.86	0.9945
4.5	*M_t_* = 0.10 *t* + 8.44	0.9121	*M_t_* = 71.60(1 − e^(^^3×10^(−3))t^)	0.9992	*M_t_* = 2.84*t*^1/2^ − 6.33	0.9827
Water	*M_t_* = 0.10 *t* + 9.47	0.9057	*M_t_* = 77.51(1 − e^(^^4×10^(−3))t^)	0.9981	*M_t_* = 3.11*t*^1/2^ − 6.78	0.9825
6.8	*M_t_* = 0.10 *t* + 3.17	0.9385	*M_t_* = 86.99(1 − e^(^^2×10^(−3))t^)	0.9886	*M_t_* = 2.94*t*^1/2^ − 11.64	0.9670
7.4	*M_t_* = 0.09 *t* + 2.30	0.9432	*M_t_* = −92470.18(1 − e^(^^1×10^(−6)^^)t^)	0.9366	*M_t_* = 2.65*t*^1/2^ − 11.00	0.9671

**Table 2:** Stability of naringin microspheres and free naringin under different conditions (*n* = 3).

Storage Condition	Time (d)	Free Naringin		Microspheres	
		Retention Rate (%)	Appearance	Retention Rate (%)	Appearance
High temperature	0	100	Light-yellow powder	100	Yellow sphere
	5	93.12 ± 1.30	Light-yellow powder	98.28 ± 0.33	Dark-yellow sphere
	10	87.70 ± 1.45	Light-yellow powder	97.54 ± 0.07	Dark-yellow sphere
High humidity	0	100	Light-yellow powder	100	Yellow sphere
	5	91.57 ± 0.03	Light-yellow powder agglomeration	97.37 ± 0.06	Yellow sphere
	10	86.87 ± 0.04	Yellow powder agglomeration	93.85 ± 0.08	Yellow sphere
Illumination	0	100	Light-yellow powder	100	Yellow sphere
	5	91.86 ± 1.22	Light-yellow powder	97.80 ± 1.37	Light-yellow sphere
	10	83.90 ± 0.99	Light-yellow powder agglomeration	94.70 ± 1.10	Light-yellow sphere

**Table 3:** Stability of naringin loaded on microspheres and free naringin under storage environments for 3 months (*n* = 3).

Time (Month)	Free Naringin		Microspheres	
	Retention Rate (%)	Appearance	Retention Rate (%)	Appearance
0	100	Light-yellow powder	100	Yellow sphere
1	92.06 ± 1.55	Light-yellow powder agglomeration	98.88 ± 1.18	Yellow sphere
3	79.36 ± 4.72	Light-yellow powder agglomeration	88.78 ± 2.85	Dark-yellow sphere

**Table 4:** Changes in yogurt pH value over time. Different superscript letters in the same row indicate significant difference (*n* = 3, *p* < 0.05).

Time (d)	CY	NY	MY
1	4.44 ± 0.01 ^b^	4.40 ± 0.01 ^c^	4.53 ± 0.01 ^a^
7	4.30 ± 0.02 ^b^	4.27 ± 0.00 ^b^	4.45 ± 0.01 ^a^
14	4.12 ± 0.01 ^b^	4.10 ± 0.01 ^b^	4.39 ± 0.02 ^a^
**Decline rate (%)**	7.14 ± 0.27 ^A^	6.89 ± 0.45 ^A^	3.02 ± 0.38 ^B^

## Data Availability

Data are contained within the article and supplemental materials.

## Supplementary Materials

The following supporting information can be downloaded at: https://www.mdpi.com/article/10.3390/foods11142147/s1, Table S1: The composition of dissolution medium.
