# Supplementary material for: Preparation, Physicochemical Characterization, and Antioxidant Activity of Naringin–Silk Fibroin–Alginate Microspheres and Application in Yogurt
Source: Foods. 2022 Jul 20;11(14):2147. doi: 10.3390/foods11142147 (PMC9318321; doi:10.3390/foods11142147)
Supplement: Supplementary file 1 [file foods-11-02147-s001.zip › foods-1782407-supplementary.pdf]

Table S1. The composition of dissolution medium.

pH	Composition			
	HCl (μL/mL)	KH_2PO_4 (mg/mL)	Na_2HPO_4 (mg/mL)	NaOH (mg/mL)
1.2	9			
4.5		6.8		
6.8		3.4	3.55	
7.4		6.8		1.58
